# Design, Synthesis and Anticancer Evaluation of Substituted Cinnamic Acid Bearing 2-Quinolone Hybrid Derivatives

**DOI:** 10.3390/molecules26164724

**Published:** 2021-08-04

**Authors:** Ali H. Abu Almaaty, Nermeen A. Elgrahy, Eman Fayad, Ola A. Abu Ali, Ahmed R. E. Mahdy, Lamiaa A. A. Barakat, Mohammed El Behery

**Affiliations:** 1 Zoology Department, Faculty of Science, Port Said University, Port Said 42526, Egypt; ali_zoology_2010@yahoo.com; 2 Chemistry Department (The Division of Biochemistry), Faculty of Science, Port Said University, Port Said 42526, Egypt; nermeenelgrahy291@gmail.com (N.A.E.); lamiaabarakat@yahoo.com (L.A.A.B.); 3 Department of Biotechnology, Faculty of Science, Taif University, P.O. Box 11099, Taif 21944, Saudi Arabia; e.esmail@tu.edu.sa; 4 Department of Chemistry, College of Science, Taif University, P.O. Box 11099, Taif 21944, Saudi Arabia; O.abuali@tu.edu.sa; 5 Chemistry Department (The Division of Organic Chemistry), Faculty of Science, Port Said University, Port Said 42526, Egypt; ahmed.ragab@sci.psu.edu.eg

**Keywords:** 2-quinolone hybrid derivatives, cytotoxicity, apoptosis, cell cycle analysis, topoisomerase II

## Abstract

A new series of hybrid molecules containing cinnamic acid and 2-quinolinone derivatives were designed and synthesized. Their structures were confirmed by ^1^H-NMR, ^13^C-NMR and mass analyses. All the synthesized hybrid molecules were assessed for their in vitro antiproliferative activity against more than one cancer cell lines. Compound 3-(3,5-dibromo-7,8-dihydroxy-4-methyl-2-oxoquinolin-1(2*H*)-ylamino)-3-phenylacrylic acid (**5a**) with IC_50_ = 1.89 μM against HCT-116 was proved to the most potent compound in this study, as compared to standard drug staurosporin. DNA flow cytometry assay of compound **5a** revealed G2/M phase arrest and pre-G1 apoptosis. Annexin V-FITC showed that the percentage of early and late apoptosis was increased. The results of topoisomerase enzyme inhibition activity showed that the hybrid molecule **5a** displays potent inhibitory activity compared with control.

## 1. Introduction

Molecular hybridization is a commonly molecular modification approach to obtain multiple compounds with therapeutic advantages over the two different drugs [1,2,3,4,5,6,7,8]. The novel hybrid molecules have the potential to enhance efficacy, improve safety, be cost-effective and reduce the propensity to elicit resistance relative to the parent drugs [9,10]. It is therefore understandable that the investigation of new hybrid anticancer drugs has recently become of great therapeutic interest.

2-quinolones (1-azacoumarins) play an important role in anticancer drug design since their derivatives have shown excellent anticancer activity through different mechanisms such as growth inhibition via apoptosis or inhibition of angiogenesis [11,12,13,14]. Additionally, the antineoplastic activity of these quinolones was attributed to intercalating binding to DNA [15]. Moreover, 6-substituted-1-azacoumarins are under human clinical experiments as an effective oral antitumor medicine [16]. Furthermore, subsequent, introduction of hydrophilic side chain to 2-quinolone scaffold led to the discovery of topotecan and irinotecan which currently used as anticancer drugs [17].

Cinnamic acid and its natural analogues are known for the treatment of cancer for over centuries [18,19]. The hydroxy cinnamic acids are natural products arisen from the deamination of the phenyl alanine. Natural hydroxy cinnamates are extremely potent antitumor agents [20,21,22]. Chemically, it is an aromatic fatty acid composed of a phenyl ring substituted with an acrylic acid group, commonly in the trans-geometry, and has low toxicity in human exposure. Cinnamic acid derivatives have been evaluated as pharmacologically active compounds [23]. They show a remarkable variety of biological activities and are often used as promising starting compounds for the development of new, highly effective drugs [24,25,26]. Cinnamic acid possesses α,β unsaturated carbonyl moiety, which can be considered as a Michael acceptor, an active moiety often employed in the design of anticancer drugs [27].

**Figure 1:**
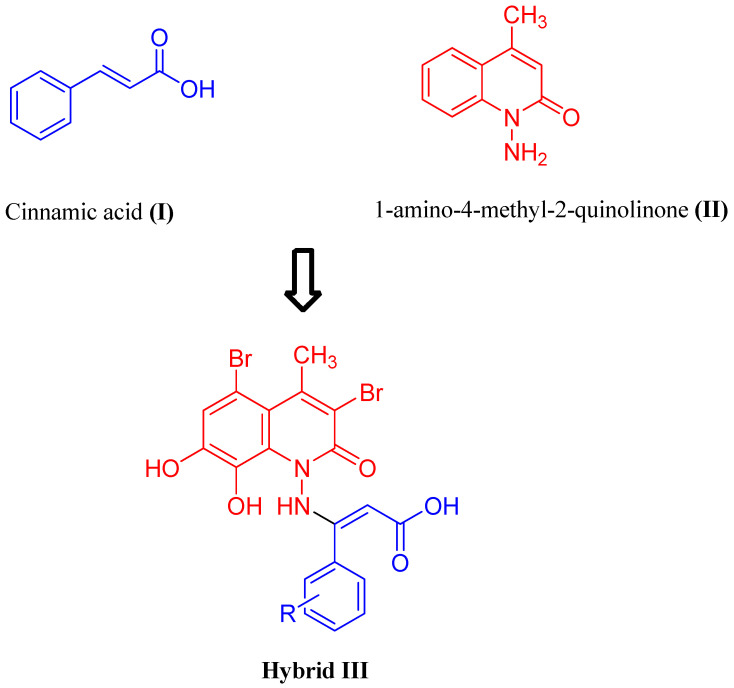
Molecular strategy related to cinnamic acid-2-quinolone hybrid **III** obtained from hybridization of cinnamic acid (**I**) and 1-amino-4-methyl-2-quinolinone (**II**).

Based on the potent anticancer activity displayed by known quinolone derivatives, a new series of hybrid molecules were designed and synthesized. The hybrid structure contains cinnamic acids bearing 7,8-dihydroxy-4-methyl-1-amino-2-quinolinone as shown in Figure 1.

## 2. Results and Discussion

### 2.1. Chemistry

**Scheme 1:**
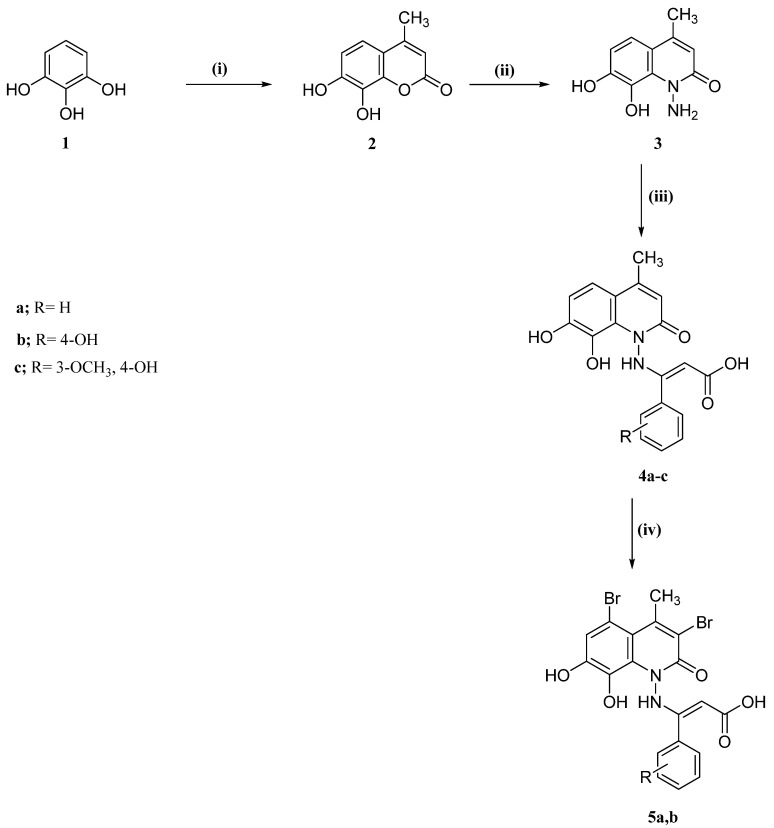
Synthesis of 3-(2-oxoquinolin-1(2*H*)-ylamino)-3-arylacrylic acid (**4** and **5**). Reagents and reaction condition: (**i**) CH_3_COCH_2_COOEt, conc H_2_SO_4_, (**ii**) N_2_H_4_, pyridine; (**iii**) Ar-CH=CH-COOH, NaOAc, (**iv**) Br_2_, AcOH.

The synthetic pathway leading to the 7,8-dihydroxy-4-methyl-1-amino-quinoline-2-one (**3**) and 3-(2-oxoquinolin-1(2*H*)-ylamino)-3-arylacrylic acid derivatives **4** and **5** is outlined in Scheme 1.

7,8-dihydroxy-4-methylcoumarin (**2**) was obtained via the condensation of pyrogallol with ethyl acetoacetate in the presence of acid catalyst according to literature procedure [28]. Reaction of 7,8-dihydroxy-4-methyl-coumarin (**2**) with hydrazine hydrate in pyridine led to the formation of 1-amino-7,8-dihydroxy-4-methylquinolin-2(1*H*)-one (**3**). FT-IR spectrum of compound **3** showed the absence absorption band of carbonyl function for the coumarin ring, in addition to new absorption band at 1688 cm^−1^ due to carbonyl function of the amide group, in addition two new bands at 3225, and 3178 cm^−1^ related to NH_2_ group. ^1^H-NMR spectrum of compound **3** showed characteristic two singlet signals at δ 2.35 and 6.12 ppm due to the protons of methyl group (CH_3_) and H-3 of quinolinone ring. Protons of the aromatic ring were appeared at δ 6.82 and 7.10 as doublet signal of H-5 and H-6 for the quinolinone ring. The ^13^C-NMR spectrum of compound **3** showed four carbon signals at δ 160.73, 154.44, 150.01 and 143.75 ppm assigned to carbonyl function (C=O), two carbons (C-O) and one carbon (C-N) groups.

Treatment of 1-amino-7,8-dihydroxy-4-methylquinolin-2(1*H*)-one (**3**) with substituted cinnamic acids in reflexing ethanol in the presence of fused sodium acetate provided the corresponding 3-(7,8-dihydroxy-4-methyl-2-oxoquinolin-1(2*H*)-ylamino)-3-arylacrylic acid **4a**–**c**.

The structure of compounds **4a**–**c** has been described by IR, ^1^H-NMR and ^13^C-NMR. The IR spectrum of compound **4a** as representative example, revealed the presence of two stretching vibration bands for NH and carbonyl of acid at 3232 and 1695 cm^−1^, respectively. The ^1^H-NMR spectrum of compound **4a** showed additional signals in the region δ 6.52–7.69 ppm with the presence of aromatic, olefinic and NH protons. In addition, the ^13^C-NMR spectrum of compound **4a** showed three signals at δ 168.31, 160.74 and 18.73 ppm assigned to the carbons of two carbonyl and methyl groups, respectively. In addition, the ^13^C-NMR spectrum of compound **4a** displayed an additional carbon signals in the region of δ 134.77–110.62 ppm attributed to the carbons of quinoline, aromatic and olefinic carbons.

The halogenation of compounds **4a,b** with bromine in glacial acetic with stirring at 60 °C led to the formation of 3-(3,5-dibromo-7,8-dihydroxy-4-methyl-2-oxoquinolin-1(2*H*)-ylamino)-3-arylacrylic acid **5a,b**. In the ^1^H-NMR spectrum of compound **5a** Protons of the aromatic and H-6 of quinolinone ring were observed as multiplet signals within the expected chemical shift region at δ 7.35–7.69 ppm. The ^13^C-NMR spectrum of **5a** showed four carbons appeared as four signals at δ 152.09, 147.53, 144.41 and 141.54 ppm due to two carbons of C-O and C-N groups. In addition, carbons of the aromatic and quinolinone rings were observed a characteristic carbon signals in the region δ 138.68–107.12 ppm.

### 2.2. Evaluation of Biological Activity

#### 2.2.1. In Vitro Cytotoxic Activity against Three Cancer Cell Line

The effect of quinolone derivatives **3**, **4a,b** and **5a**,**b** on the viability of three cancer cell lines were studied using MTT assay (Table 1). The cytotoxicity was assessed using Staurosporin (STU) as positive control. The three cancer cell lines are MCF-7 (breast cancer cell line), HepG2 (hepatocellular carcinoma cell line) and colon carcinoma (HCT-116). The obtained results indicated that compound **5b** exhibited the most potent cytotoxic activity against MCF-7 cells with IC_50_ value 8.48 μM. In addition, compound **5a** exhibited potential cytotoxic activity against HepG2 and HCT-116 cells with IC_50_ values 4.05 and 1.89 μM, respectively. From the obtained results it can be concluded that, the presence of bromine at C3 and C5 of quinolone ring has better anticancer activity against three cancer cell lines.

#### 2.2.2. Cell Cycle Analysis

**Figure 2:**
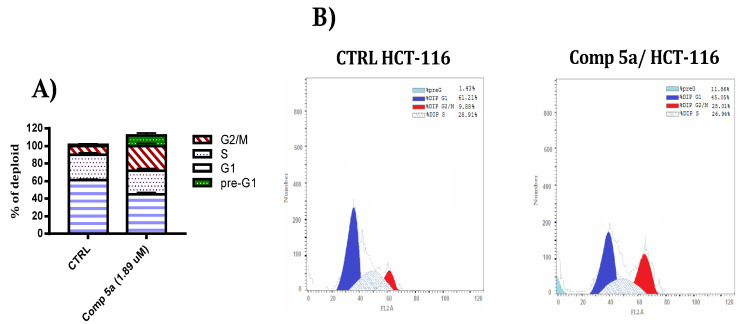
(**A**) Graphical representation of cell cycle distribution of HCT-116 cell line induced by compound **5a**. (**B**) Cell cycle distribution of HCT-116 cell line induced by compound **5a**.

Targeting the cell cycle of tumor cells has been recognized as a promising strategy for cancer therapy [29,30]. In this study, cell cycle analysis was followed via DNA flow cytometric analysis using propidium iodide (PI) as staining agent following hybrid molecule **5a** treatment at its IC_50_ concentration for 48 h. The results showed that the tested compound increase the percentage of cell population at G2/M phase by 2.83-fold compared to untreated control group. While the number of cells in the G1 and S phases were reduced. Additionally, the result showed that compound **5a** increased the percentage of cells at pre-G1 phase by 8.29-fold compared to the control (Figure 2).

#### 2.2.3. Apoptosis Detection Assay

**Figure 3:**
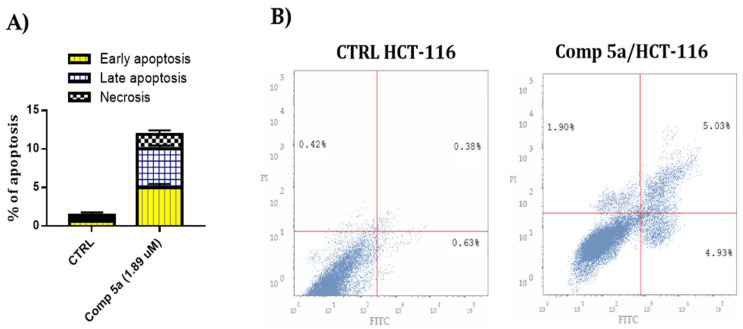
(**A**) Graphical representation of apoptosis effect of compound **5a** on HCT-116 cell line. (**B**) Apoptosis effect of compound **5a** on HCT-116 cell line.

To characterize the mode of cell death caused by compound **5a**, a biparametric cytofluorimetric analysis using PI and Annexin-V-FITC was performed in HCT-116 cells after treatment with compound **5a** at its IC_50_ concentration dose value for 48 h (Figure 3). The results revealed that compound **5a** increases in the percentage of total apoptosis compared with untreated control group. The percentage of early apoptosis was increased from 0.71% to 6.65% compared with the untreated control group. Additionally, compound **5a** can increase the percentage of late apoptosis from 0.52% to 7.87% compared with untreated control.

#### 2.2.4. Topoisomerase II Inhibitory Activity

**Figure 4:**
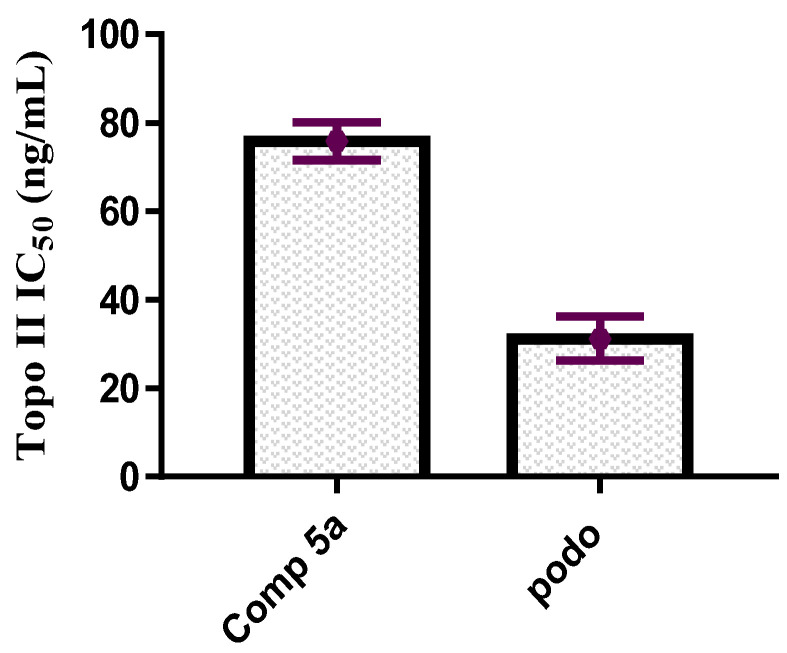
Graphical presentation for comparison of topo II IC_50_ (ng/mL) of the compound **5a** and podo.

Compound **5a** was evaluated for inhibitory activity on Topoisomerase II (topo II). Five dose concentrations were used and IC_50_ concentration was determined. Podophyllotoxin (podo) was employed as the standard. It is observed that, compound **5a** revealed strong inhibitory activity against topo II with IC_50_ value of 75.82 ng/mL, compared to the reference compound podo (IC_50_ = 31.24 ng/mL) (Figure 4).

### 2.3. Molecular Docking Study

**Figure 5:**
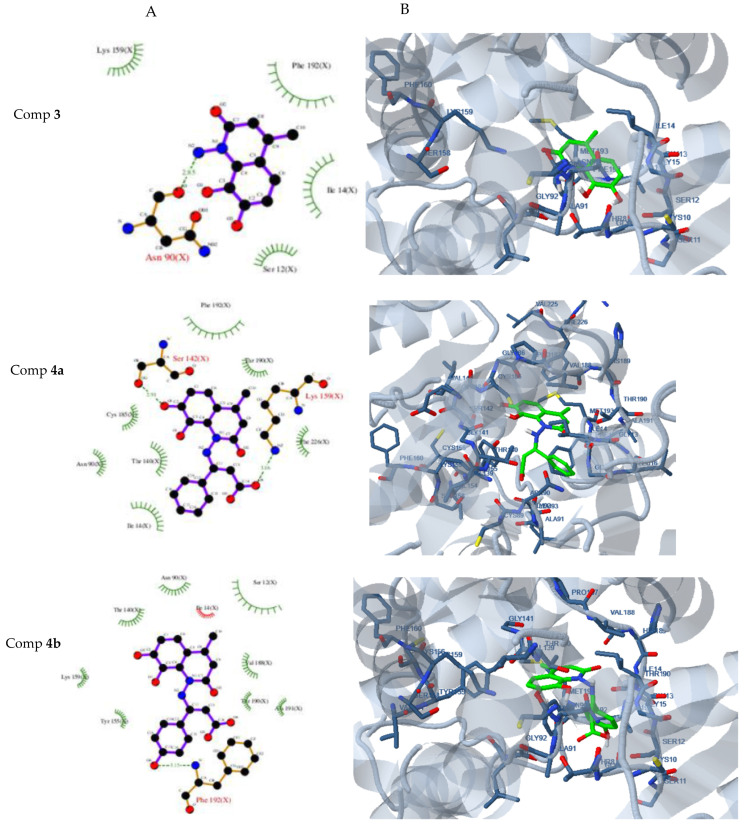
Interaction of docked compounds **3**, **4a** and **4b** inside the binding site of (3HB5): (**A**) 2D interaction; (**B**) 3D interaction.

**Figure 6:**
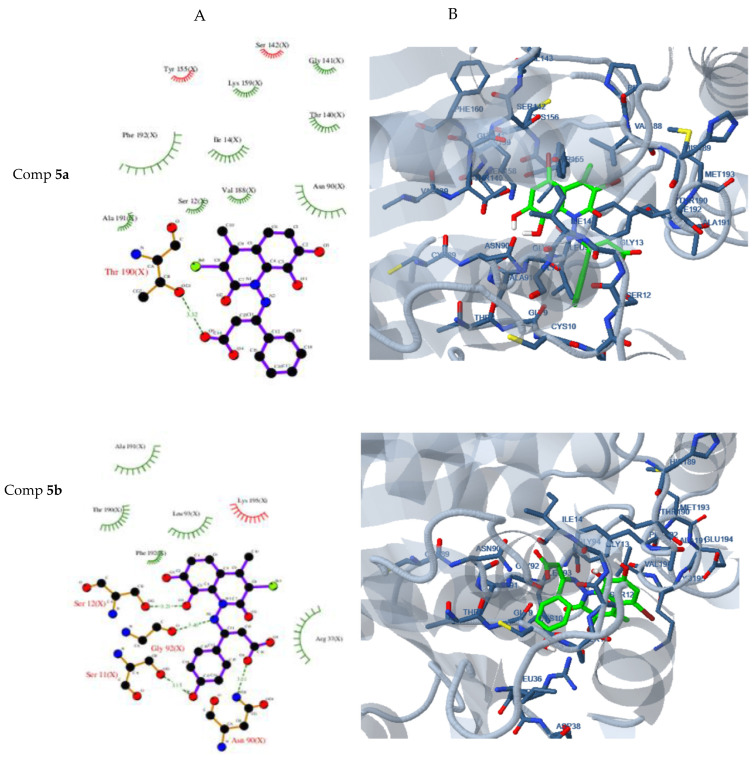
Interaction of docked compounds **5a** and **5b** inside the binding site of (3HB5). (**A**) 2D interaction; (**B**) 3D interaction.

Molecular docking is a vital tool in drug design [31,32,33,34]. Molecular docking achieves a confirmation for the protein and ligand interactions. In this work, molecular docking was conducted using the synthesized compounds (**3**, **4a**,**b**, and **5a**,**b**) against breast cancer protein (PDB: 3HB5). The docking results showed a potential structure-activity relationship between the target compounds (**3**, **4a**,**b**, and **5a**,**b**) against 3HB5 protein as shown in (Table 2, Figure 5 and Figure 6). Compounds **4a** and **5a** showed the highest binding interaction against the key amino acids of the 3HB5 with docking scores −9.8 and −9.56 kcal.mol^−1^ respectively. The binding affinity and interaction bonds between docked compounds and active site of target protein 3HB5 were hydrogen bond, polar and hydrophobic interaction as shown in (Table 2). Compounds **5a** exhibited a potential interaction toward (3HB5) receptor which is compatible with cytotoxicity and biological results.

## 3. Material and Methods

### 3.1. Chemistry

The melting point was measured on electro thermal 200 digital melting point device and was uncorrected. The ^1^H and ^13^C-NMR spectra were measured with a Bruker Avance 400 MHz spectrometer (Chichago, Elk Grove Village, USA) using the DMSO-*d_6_* as solvent. The IR data were obtained with a shimadzu 470 spectrometer (Kyoto, Japan). The molecular weight of the synthesized compounds was determined by electron ionization (EI) mass spectrometer performed using a probe Agilent MSD 5975 spectrometer (Agilent Technologies, Inc. 5301 Stevens Creek Boulevard Santa Clara, CA, USA) operating at 70 eV. The elemental analysis was performed on a Perkin-Elmer 2400 series CHN elemental analyzer (Haan, Germany) chemical and reagents were purchased from either Aldrich or Sigma and all reagents were analytical grade.

#### 3.1.1. Synthesis of 7,8-Dihydroxy-4-methyl Coumarin (**2**)

A mixture of pyrogallol (0.01 mol) and ethyl acetoacetate (0.01 mol) in the presence of concentrated sulfuric acid (2 mL) as acid catalyzed was heated under reflux on a water bath for 2 h. The reaction mixture was cooled and poured into water, the solid formed was filtered off, washed with water and dried. Finally, the product was crystallized from hot water.

7,8-Dihydroxy-4-methyl coumarin (**2**, known). IR (KBr) ν_max_: 3458 (br. OH), 1725 (C=O), 1605, 1583 (C=C), 1170, 1065, 1035 (C-O) cm^−1^. ^1^H-NMR (DMSO-d_6_) δ: 2.34 (s, 3H, CH_3_), 6.21 (s, 1H, H-3 of coumarin ring), 6.91 (d, 1H, H-6 of coumarin ring), 7.21 (d, 1H, H-5 of coumarin ring), 9.68 (br. s, 1H, OH), 9.14 (br. s, 1H, OH) ppm. MS (*m/z*, %) = 192 (M^+^, 76.30). Anal. Calcd. for C_8_H_8_O_4_ (192): C, 62.50; H, 4.17. Found: C, 62.33; H, 4.03.

#### 3.1.2. Synthesis of 1-Amino-7,8-dihydroxy-4-methylquinolin-2(1H)-one (**3**)

A solution of 7,8-dihydroxy-4-methyl coumarin (0.01 mol) in pyridine (30 mL) was added hydrazine hydrate (0.02 mol), then heating under reflux for 6 h. The reaction mixture was cooled, poured into ice-water and neutralized with dilute hydrochloric acid (1N). The solid obtained was filtered off, washed with water, dried and purified by crystallization from absolute ethanol.

1-Amino-7,8-dihydroxy-4-methylquinolin-2(1H)-one (**3**) as yellow crystals, yield 71%, m.p. 265–267 °C. IR (K Br) ν_max_: 3418 (br OH), 3225, 3178 (NH_2_), 1648 (C=O), 1619, 1587 (C=C), 1061, 1006 (C-O) cm^−1^. ^1^H-NMR (DMSO-*d_6_*) δ: 2.35 (s, 3H, CH_3_), 4.12 (br.s, 2H, NH_2_), 6.12 (s, 1H, H-3 of quinolinone), 6.82 (d, 1H, H-6 of quinolinone), 7.09 (d, 1H, H-5 of quinolinone) ppm. ^13^C-NMR (DMSO-*d_6_*) δ: 160.73 (C-2), 154.44 (C-7), 150.01 (C-8), 143.75 (C-9), 132.69 (C-4), 115.92 (C-10), 113.17 (C-5), 112.60 (C-6), 110.59 (C-3), 18.73 (C-11) ppm. MS (*m/z*, %) = 206 (M^+^, 17.30). Anal. Calcd. for C_10_H_10_N_2_O_2_ (206): C, 58.25; H, 4.85; N, 13.59. Found: C, 58.09; H, 4.66; N, 13.39.

#### 3.1.3. Synthesis of 3-(7,8-Dihydroxy-4-methyl-2-oxoquinolin-1(2H)-ylamino)-3-arylacrylic Acid (**4a**–**c**)

A suspension of a suitable cinnamic acid derivative (0.01 mol) and 1-amino-7,8-dihydroxy-4-methylquinolin-2(1*H*)-one (**3**, 0.01 mol) in ethanol (30 mL), in the presence of fused sodium acetate (0.03 mol) was refluxed for 4 h. The reaction mixture was cooled down, poured into water, and the solid formed was filtered. The product was crystallized from a suitable solvent to give compound **4a**–**c**.

3-(7,8-dihydroxy-4-methyl-2-oxoquinolin-1(2H)-ylamino)-3-phenylacrylic acid (**4a**) Pale yellow crystals, yield 76%, m.p. 185–187 °C. Crystallized from ethanol/H_2_O (3:1). IR (KBr) ν_max_: 3550–3350 (br. OH), 3232 (NH), 1705–1675 (br. C=O), 1629, 1584 (C=C), 1229, 1061, 1006 (C-O) cm^−1^. ^1^H-NMR (DMSO-*d_6_*) δ: 2.36 (s, 3H, CH_3_), 6.13 (s, 1H, H-3 of quinolinone), 6.56 (s, 1H, H-olefinic of cinnamic acid), 6.38 (d, 1H, H-6 of quinoline ring), 7.10 (d, 1H, H-5 of quinolinone ring), 7.41–7.69 (m, 6H, Ar-H and NH) ppm. ^13^C-NMR (DMSO-*d_6_*) δ: 168.31 (C-21), 160.74 (C-7),150.00 (C-8), 144.11 (C-9), 143.82 (C-13), 134.77 (C-17), 132.72 (C-4), 130.62 (C-14), 129.37 (C-15,9), 128.63 (C-16,18), 120.09 (C-20), 115.92 (C-10), 113.21 (C-5), 112.67 (C-6), 110.62 (C-3), 18.73 (C-H) ppm. MS (*m/z*, %) = 352 (M^+^, 13.51), Anal. Calcd. for C_19_H_16_N_3_O_5_ (352), C, 64.77; H, 4.54; N, 7.95. Found: C, 64.58; H, 4.33; N, 7.77.

3-(7,8-Dihydroxy-4-methyl-2-oxoquinolin-1(2H)-ylamino)-3-(4-hydroxyphenyl)acrylic acid (**4b**) Pale yellow crystals, yield 69%, m.p. 181–183 °C. Crystallized from ethanol/H_2_O (3:1). IR (KBr) ν_max_: 3560–3100 (br. OH), 3232 (NH), 1701–1660 (C=O), 1626, 1593 (C=C), 1170, 1062, 1007 (C-O) cm^−1^. ^1^H-NMR (DMSO-*d_6_*) δ: 2.35 (s, 3H, CH_3_), 6.12 (s, 1H, H-3 of quinolinone ring), 6.28–7.53 (m, 8H, Ar-H, olefinic and NH) ppm. ^13^C-NMR (DMSO-*d_6_*) δ: 168.45 (C-21), 160.70 (C-2), 160.70 (C-17), 154.39 (C-7), 149.87 (C-8), 144.65 (C-9), 143.79 (C-13), 132.63 (C-4), 130.56 (C-15,19), 125.74 (C-14), 116.22 (C-16,18), 115.95 (C-10), 115.81 (C-20), 113.24 (C-5), 112.60 (C-6), 110.66 (C-3), 18.71 (C-11) ppm. MS (*m/z*, %) = 368 (M^+^, 11.51). Anal. Calcd. for C_19_H_16_N_2_O_6_ (368): C, 61.96; H, 3.35; N, 7.61. Found: C, 61.73; H, 3.11; N, 7.51.

3-(7,8-Dihydroxy-4-methyl-2-oxoquinolin-1(2H)-ylamino)-3-(4-hydroxy-3-methoxyphenyl)acrylic acid (**4c**). Pale yellow crystals, yield 67%, m.p. 175–177 °C. Crystallized from DMF/H_2_O (1:1). IR (KBr) ν_max_: 3550–2984 (br. OH), 3232 (NH), 1700–1652 (C=O), 1620, 1595 (C=C), 1172, 1141, 1062 (C-O) cm^−1^. ^1^H-NMR (DMSO-*d_6_*) δ: 2.35 (s, 3H, CH_3_), 3.82 (s, 3H, OCH_3_), 6.12 (s, 1H, H-3 of quinolinone ring), 6.36–7.52 (m, 7H, Ar-H, H-olefinic and NH), 9.35 (br.s, 1H, OH), 10.02 (br.s, 2H, 2xOH) ppm. ^13^C-NMR (DMSO-*d_6_*) δ: 168.51 (C-21), 160.70(C-2), 154.41 (C-7), 149.87 (C-8), 149.53 (C-17), 148.37 (C-18), 144.99 (C-9), 143.78 (C-13), 132.26 (C-4), 126.23 (C-15), 123.21 (C-19), 116.10 (C-10), 115.95 (C-14,16), 113.23 (C-5), 112.58 (C-6), 111.56 (C-20), 110.65 (C-3), 56.13 (C-22), 18.72 (C-11) ppm. MS (*m/z*, %) = 398 (M^+^, 8.60). Anal. Calcd. for C_20_H_18_N_2_O_7_ (398): C, 60.30; H, 4.52; N, 7.03. Found: C, 60.11; H, 4.29; N, 6.89.

#### 3.1.4. Synthesis of 3-(3,5-Dibromo-7,8-dihydroxy-4-methyl-2-oxoquinolin-1(2H)-ylamino)-3-arylacrylic Acid (**5a**,**b**)

In 15 mL of glacial acetic acid, compound **4** (0.01 mol) was dissolved. Then, 10 mL of bromine (0.02 mol) in glacial acetic acid was added dropwise to compound **4** solution with stirring at 60 °C. After 5–10 min. the bromine color was discharged and a yellow solution remained. At this point, an additional 0.5–1 mL of the bromine-AcOH solution was added with stirring at room temperature for 30–45 min. The reaction mixture was poured into water and the resulting product was filtered off, washed with water and dried. Finally, The product was crystallized from DMF/H_2_O (1:1) to give **5a**,**b**.

3-(3,5-Dibromo-7,8-dihydroxy-4-methyl-2-oxoquinolin-1(2H)-ylamino)-3-phenylacrylic acid (**5a**) Yellow crystals, yield 71%, m.p. 225–227 °C. IR(KBr) ν_max_: 3452 (br. OH), 3254 (NH),1710–1705 (C=O), 1627, 1604, 1558 (C=C), 1223, 1072, 1026 (C-O) cm^−1^. ^1^H-NMR (DMSO-*d_6_*) δ: 2.55 (s, 3H, CH_3_), 5.32 (d, 1H, H-olefinic) 5.54 (d, 1H, H-olefinic of two isomer), 6.54 (d, 1H, NH), 7.38–7.69 (m, 6H, Ar-H for two isomer) 10.35 (br.s, 1H, OH), 10.53 (br.s, 1H, OH) ppm. ^13^C-NMR (DMSO-*d_6_*) δ: 169.56, 168.05 (C=O of acid for two isomer), 156.46 (C-2), 152.09, 147.5, (C-O), 144.41, 141.54 (C-N), 138.68, 134.67, 133.30,130.68, 129.54, 129.35, 129.15, 128.65, 128.65, 119.66, 119.38, 113.62, 109.24, 107.12 (C-aromatic acid Quinolinone ring), 52.38, 47.69 (C-olefinic), 20.01 (CH_3_) ppm. MS (*m/z*, %) = 508(M^+^, unstable). Anal. Calcd. for C_19_H_14_N_2_Br_2_O_5_ (508): C, 44,88, H, 2.75; N, 5.51. Found: C, 44.64; H, 2.52; N, 5.33.

3-(3,5-dibromo-7,8-dihydroxy-4-methyl-2-oxoquinolin-1(2H)-ylamino)-3-(4-hydroxyphenyl)acrylic acid (**5b**). Pale yellow crystals, yield 69%, m.p. 233–235 °C. IR (KBr) ν_max_: 3580–2955 (br. OH and NH), 1717–1705 (br. C=O), 1607, 1558 (C=C), 1239, 1160, 1101, 1028 (C-O) cm^−1^. ^1^H-NMR (DMSO-d*_6_*) δ: 2.53 (s, 3H, CH_3_), 7.01–8.16 (m, 6H, Ar-H and H-olefinic) ppm. ^13^C-NMR (DMSO-*d_6_*) δ: 166.21, 156.46 (C=O), 152.08, 151.84, 151.04 (C-O), 147.52, 141.53 (C-N), 134.49, 134.31, 133.31, 132.87, 132.46, 130.87, 130.77, 130.23, 119.36, 116.83, 113.61, 112.63, 112.34, 111.94, 109.24, 108.50, 107.78, 106.22 (C-Quinolinone, aromatic and olefinic carbons), 20.16, 20.01 (CH_3_,two isomer). MS: (*m/z*, %) = 524(M^+^, unstable). Anal. Calcd. for C_19_H_14_N_2_Br_2_O_6_ (524): C, 43.51; H, 2.67; N, 5.34. Found: C, 43.34; H, 2.42; N, 5.51. ^1^H and ^13^C NMR of compound **3**, **4a**–**c** and **5a**–**b** are available in Supplementary Materials.

### 3.2. Biological Evaluation

#### 3.2.1. Anti-Tumor Activity against Three Cancer Cell Lines

The cytotoxic activity was measured in vitro using the MTT assay. Cells were plated in 96-multiwall plate (10^5^ cells/well) for 24 h before treatment with the compounds. Test compounds were dissolved in dimethyl sulfoxide (DMSO) which did not exceed 1% final concentration. Different concentrations of the compound under test (0.39, 1.56, 6.25, 25, and 100 μg/mL) were added to the cell’s monolayer. Triplicate wells were prepared for each individual concentration. Monolayer cells were incubated with the compound(s) for 48 h at 37 °C and in atmosphere of 5% CO_2_. After 48 h, cells were fixed, washed with phosphate buffer saline (PBS, pH = 7.4) and stained with 40 μL of MTT solution (5 mg/mL of MTT in 0.9% NaCl) in each well was added and incubated for an additional 4 h. MTT crystals were solubilized by adding 180 μL of acidified isopropanol/well and the plate was shaken at room temperature, followed by photometric determination of the absorbance at 570 nm using ELISA reader. The molar concentration required to inhibit 50% of cell viability (IC_50_) was calculated and compared with the reference drug STU. The surviving fractions were expressed as means ± SD.

#### 3.2.2. Cell Cycle Analysis of Compound **5a**

HCT-116 cells, (3.0 × 10^5^ cells/well) and incubated at 37 °C for 12 h. The target cells were then treated with the compound **5a** at its IC_50_ concentration dose value for 48 h. After treatment, cells were collected and fixed with 75% ethanol at 20 °C overnight, then, cells were washed with PBS followed by centrifugation and incubated with (10 mg/mL) Rnase (Sigma, St. Louis, MO, USA) and (5 mg/mL) propidium iodide (PI, Sigma) before flow cytometry analysis (FACSCalibur cytometer using Cellquest software, BD Bioscience, San Jose, CA, USA).

#### 3.2.3. Apoptosis Determination by Annexin-V Assay

The HCT-116 cells (2 × 10^5^/well) were treated with compound **5a** at its IC_50_ concentration value for 48 h. After treatment, cells were harvested and washed twice (180 g, 10 min, 4 °C) with PBS. Each cell well was resuspended in 100 μL of binding buffer, and 5 µL Annexin V-FITC were added. After an incubation time of 10 min at room temperature, additional 400 µL of binding buffer were added for a final volume of 500 µL. Cells were stained with PI immediately before measurement. Cells were the analyzed by using *FACS Calibur* Flow cytometer (Becton and Dickinson, Heidelberg, Germany). Data thus obtained were analyzed with Cell-Quest software (Becton and Dickinson, Heidelberg, Germany).

#### 3.2.4. In Vitro Topoismerase Inhibitory Assay

Compound **5a** was selected to be evaluated against topoisomerase II [MBS#942146] using human DNA topoisomerase 2-β (TOP2β) ELISA kit according to manufacturer’s instructions. Prepare all reagents, working standards, and samples. Add 100 μL of standard and sample per well and incubate for 2 h at 37 °C. Remove the liquid of each well, do not wash. Add 100 μL of biotin-antibody to each well and incubate for 1 h at 37 °C. Aspirate each well and wash three times. Add 100 μL of horseradish peroxidase (HRP-avidin) to each well and incubate for 1 h at 37 °C. Repeat the aspiration/wash process for five times. Add 90 μL of 3,3′,5,5′-tetramethylbenzidine (TMB) substrate to each well and incubate for 15–30 min at 37 °C, protect from light. Add 50 μL of stop solution to each well and determine the optical density of each well within 5 min, using a microplate reader set to 450 nm. The values of % activity versus a series of compound concentrations (2.5, 5, 10, and 15 µM) were then plotted using non-linear regression analysis of sigmoidal dose-response curve. The IC_50_ values for compound **5a** against topoisomerase II was determined by the concentration causing a half-maximal percent activity and the data were compared with Dox as standard.

### 3.3. Molecular Docking Study

Docking calculations were estimated using a Docking Server [35]. Docking scores were examined on the 3HB5 protein model. Crucial hydrogen atoms, Kollman united atom type charges, and solvation parameters were inserted with the support of AutoDock tools. Affinity (grid) maps of 61 × 61 × 61 points and 0.375 Å spacing were created using the Autogrid program [36]. AutoDock parameter set and distance-dependent dielectric purposes were utilized in the calculation of the van der Waals and the electrostatic terms, correspondingly. Docking simulations were done via the Lamarckian genetic algorithm (LGA) and the Solis and Wets local search approach [37]. The initial position, orientation, and torsions of the ligand molecules were fixed arbitrarily. Wholly rotatable torsions were discharged throughout docking. Each docking experimentation was originated from 2 different runs that were established to end afterward a maximum of 250,000 energy evaluations. The population size was set to 150. Throughout the search, a translational step of 0.2 Å, and quaternion and torsion steps of **5** were applied.

## 4. Conclusions

In conclusion, hybrid molecules containing cinnamic acid and 2-quinolinone derivatives (**4a**–**c**) were synthesized via the reaction of cinnamic acid derivatives with 1-amino-7,8-dihydroxy-4-methylquinolin-2(1*H*)-one (**3**), which was obtainable in the reaction of 7,8-hydroxy-4-methylcoumarin (**2**) with hydrazine hydrate in pyridine. Brominated derivatives **5a**,**b** were prepared via the halogenation of compounds **4a**,**b** with bromine in glacial acetic acid. Cinnamic acids bearing 2-quinolone **4a**,**b** and their brominated derivatives **5a**,**b** were assessed for their in vitro antiproliferative activity against three cancer cell lines and the brominated derivatives were found to be more active. DNA flow cytometry assay of compound **5a** revealed G2/M phase arrest and pre-G1 apoptosis. Annexin V-FITC showed the percentage of early and late apoptosis was increased. The results of topoisomerase enzyme inhibition activity showed that the hybrid molecule **5a** display potent inhibitory activity compared with control. In conclusion, the preliminary study of the anticancer activity of the prepared compounds represents a novel strategy for the discovery of anticancer compounds which needs further investigation.

## Figures and Tables

**Table 1:** In vitro antitumor activity of quinolone derivatives **3**, **4a**,**b** and **5a**,**b** over MCF-7, HepG2 and HCT-116, cell lines. Data are expressed as the mean ± SD.

Comp No.	IC_50_ (μM)		
	MCF-7	HepG2	HCT-116
**3**	32.9 ± 1.81	8.23 ± 0.42	15.0 ± 0.79
**4a**	18.7 ± 1.03	19.4 ± 0.99	21.3 ± 1.12
**4b**	11.8 ± 0.65	15.5 ± 0.79	7.56 ± 0.4
**5a**	19.3 ± 1.06	4.05 ± 0.21	1.89 ± 0.10
**5b**	8.48 ± 0.47	30.9 ± 1.57	10.7 ± 0.57
**STU**	6.91 ± 0.38	5.23 ± 0.27	4.25 ± 0.22

**Table 2:** Docking scores of synthesized compounds (**3**, **4a**,**b**, and **5a**,**b**) against 3HB5 (PDB: 3HB5).

Comp No.	Docking Scores (kcal.mol^−1^)	Hydrogen Bonds	Polar Interaction	Hydrophobic Interactions
**3**	−6.42	ASN90	LYS159 SER12	PHE192 ILE14
**4a**	−9.81	LYS195 SER142		PHE192 ILE14 PHE226
**4b**	−7.56	PHE192	SER12 ASN90 LYS195 THR190	ILE14
**5a**	−9.56	THR190	GLY141 VAL188 SER142 TYR155	PHE192 ILE14
**5b**	−6.06	GLY92 SER12 ASN90 SER11	ARG37 THR190	ALA191

## Data Availability

Not applicable.

## Supplementary Materials

The following are available online: ^1^H and ^13^C NMR of compound **3**, **4a**–**c** and **5a**,**b**.

## Sample Availability

Samples of compounds are not available from authors.
