# Supplementary material for: Design, Synthesis and Anticancer Evaluation of Substituted Cinnamic Acid Bearing 2-Quinolone Hybrid Derivatives
Source: Molecules. 2021 Aug 4;26(16):4724. doi: 10.3390/molecules26164724 (PMC8400797; doi:10.3390/molecules26164724)
Supplement: Supplementary file 1 [file molecules-26-04724-s001.zip › molecules-1316024-supplementary.pdf]

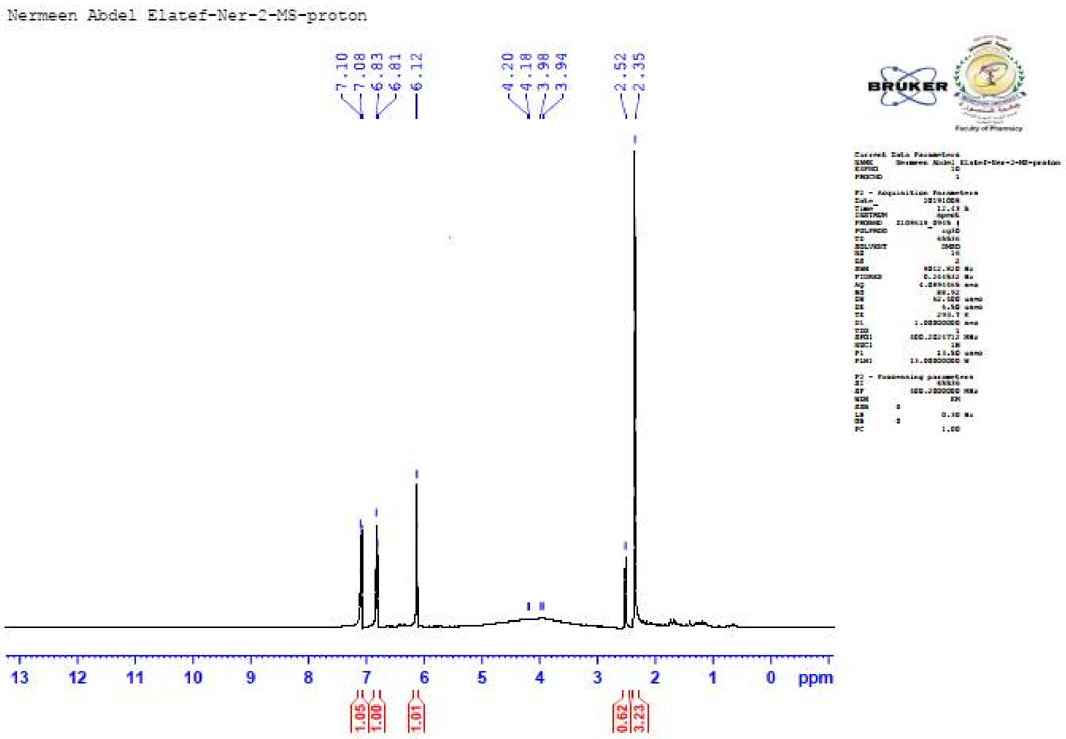

Fig. S1: ^{1}H-NMR of compound **3**

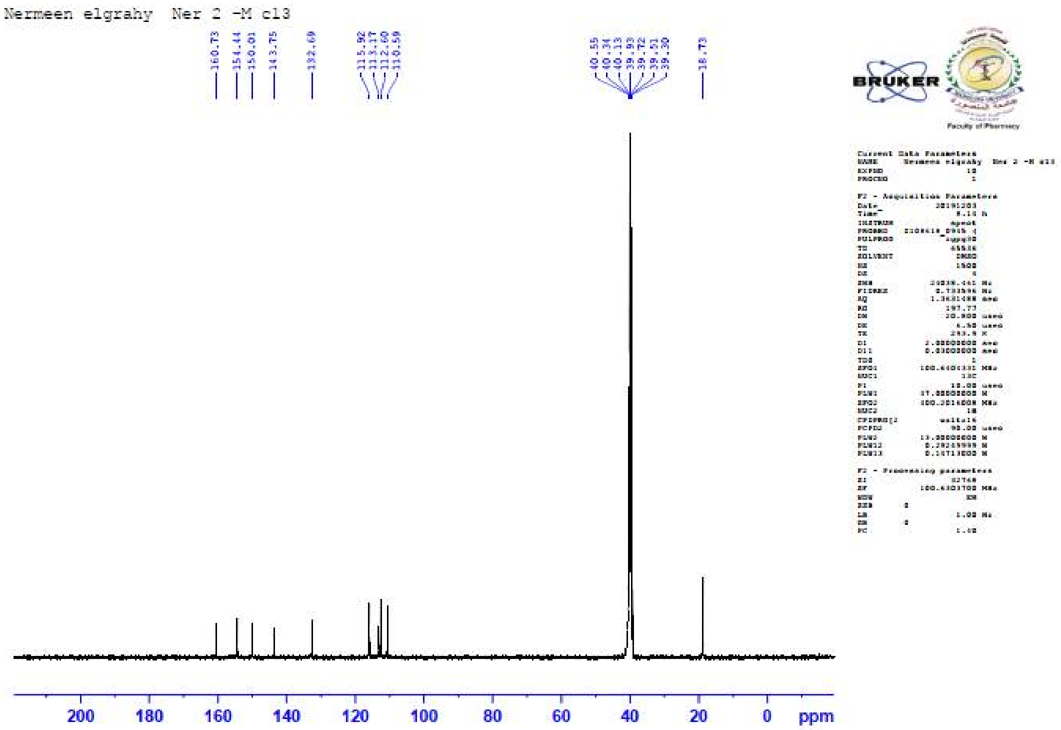

Fig. S2: ^{13}C-NMR of compound **3**

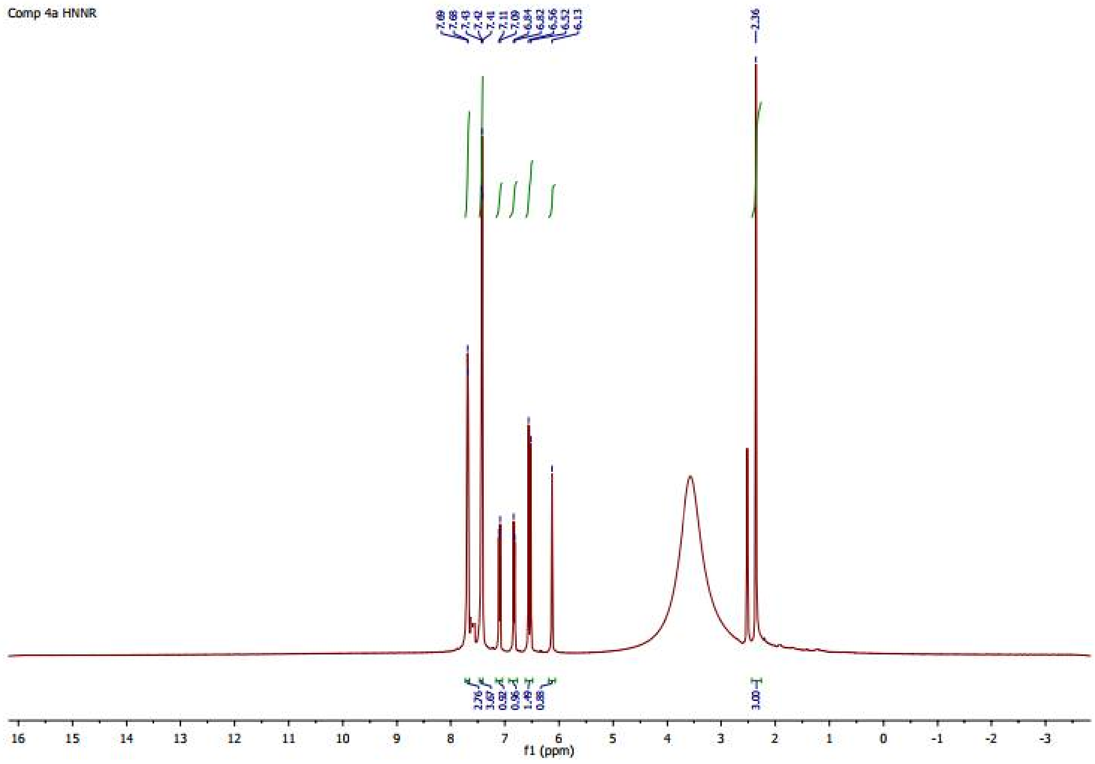

Fig. S3: ^{1}H-NMR of compound **4a**

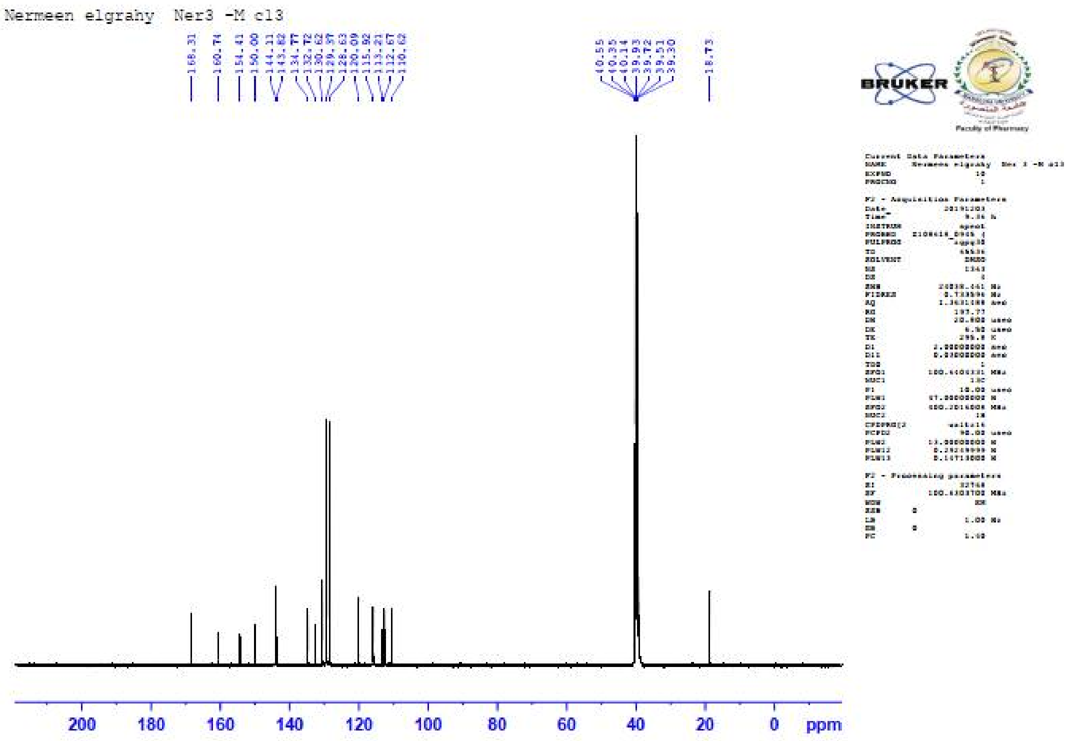

Fig. S4: ^{13}C-NMR of compound **4a**

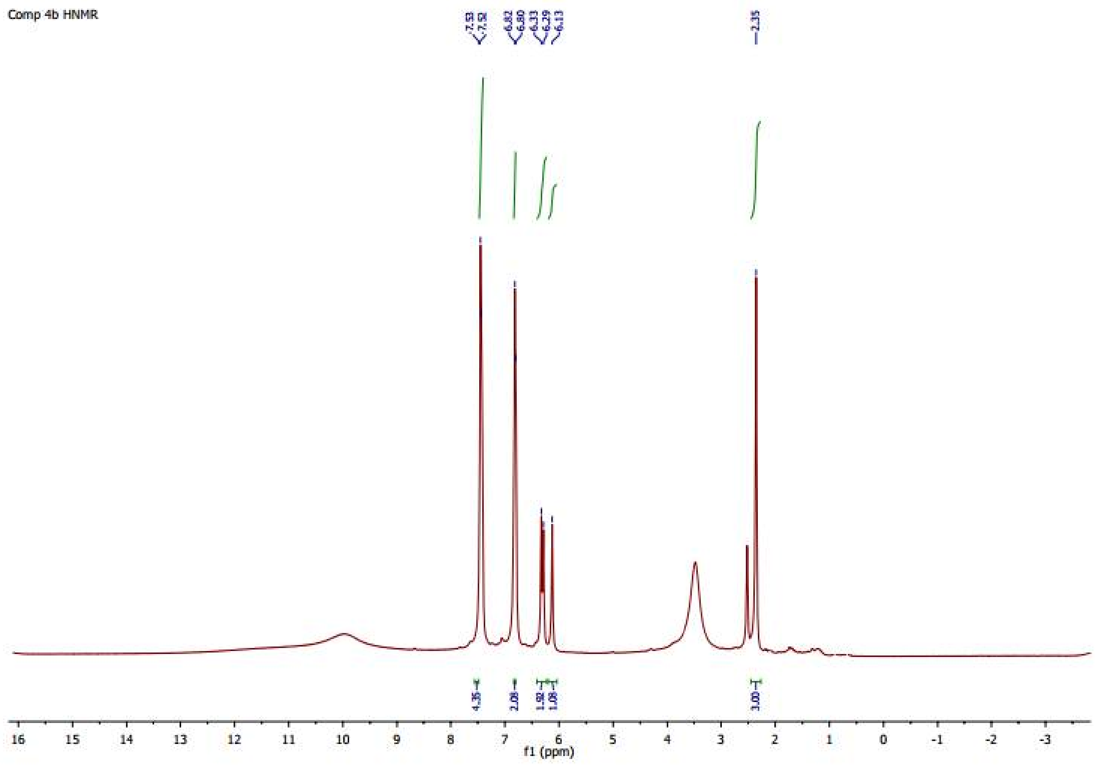

Fig. S5: ^{1}H-NMR of compound **4b**

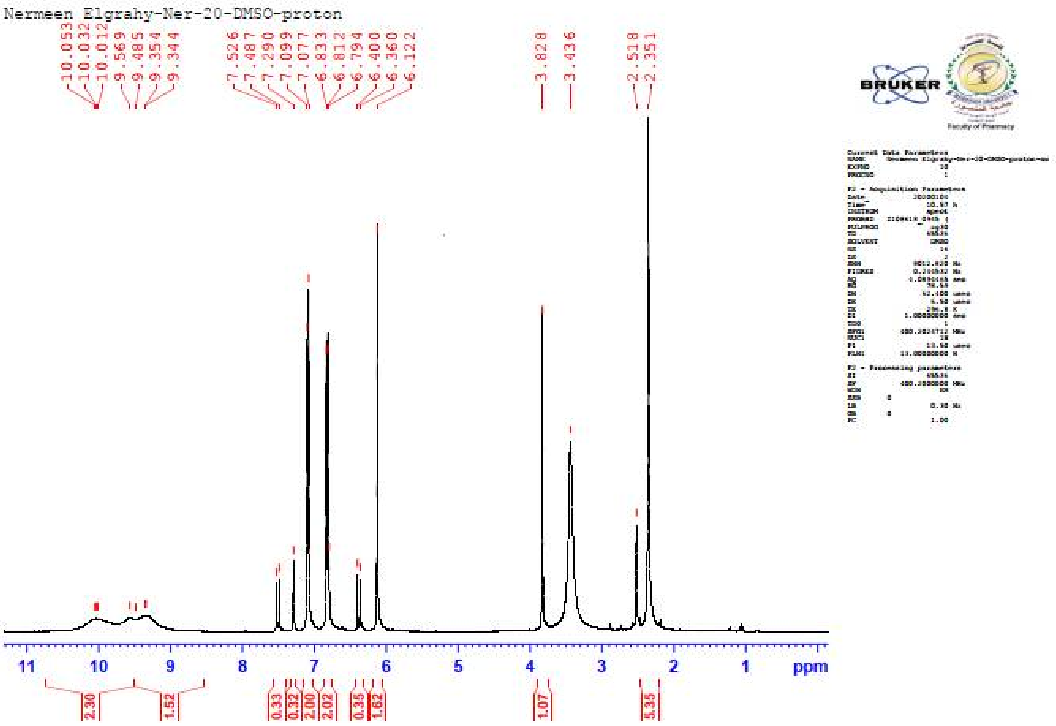

Fig. S6: ^{1}H-NMR of compound **4c**

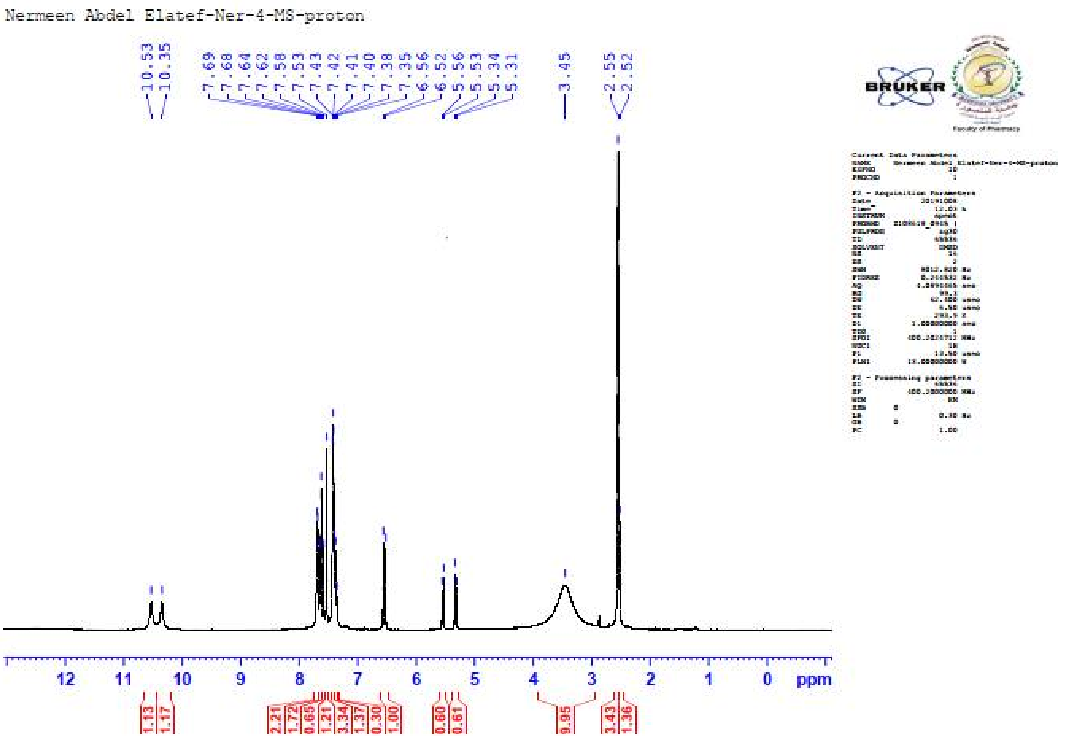

Fig. S7: ^{1}H-NMR of compound **5a**

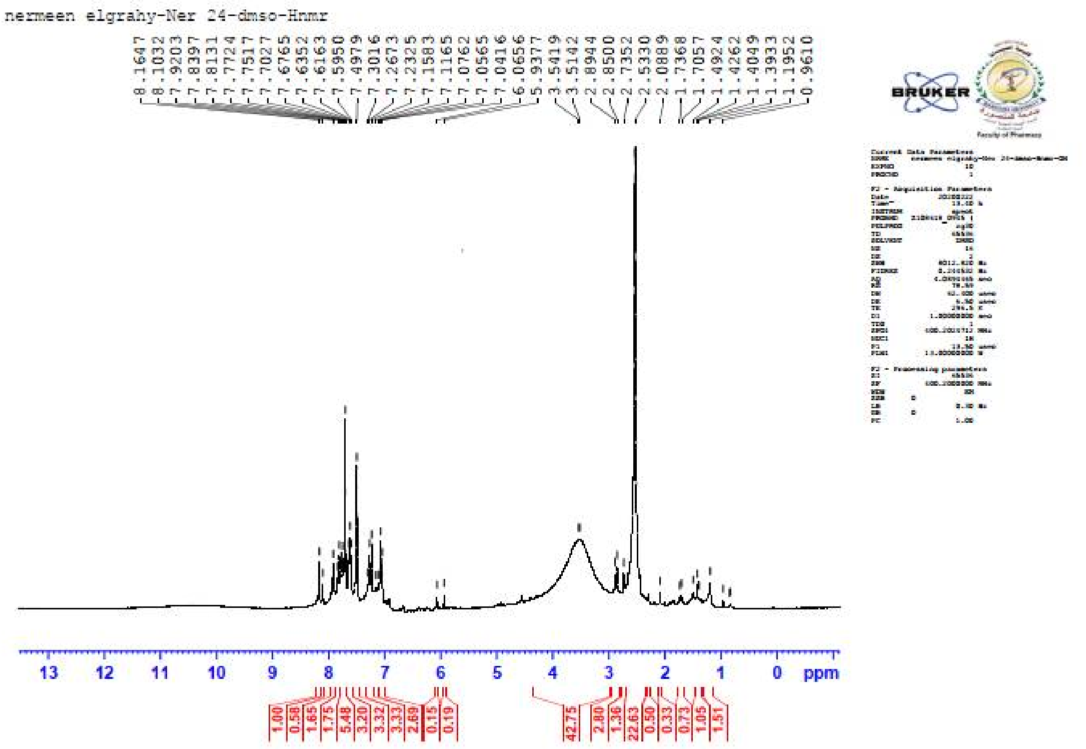

Fig. S8: ^{1}H-NMR of compound **5b**

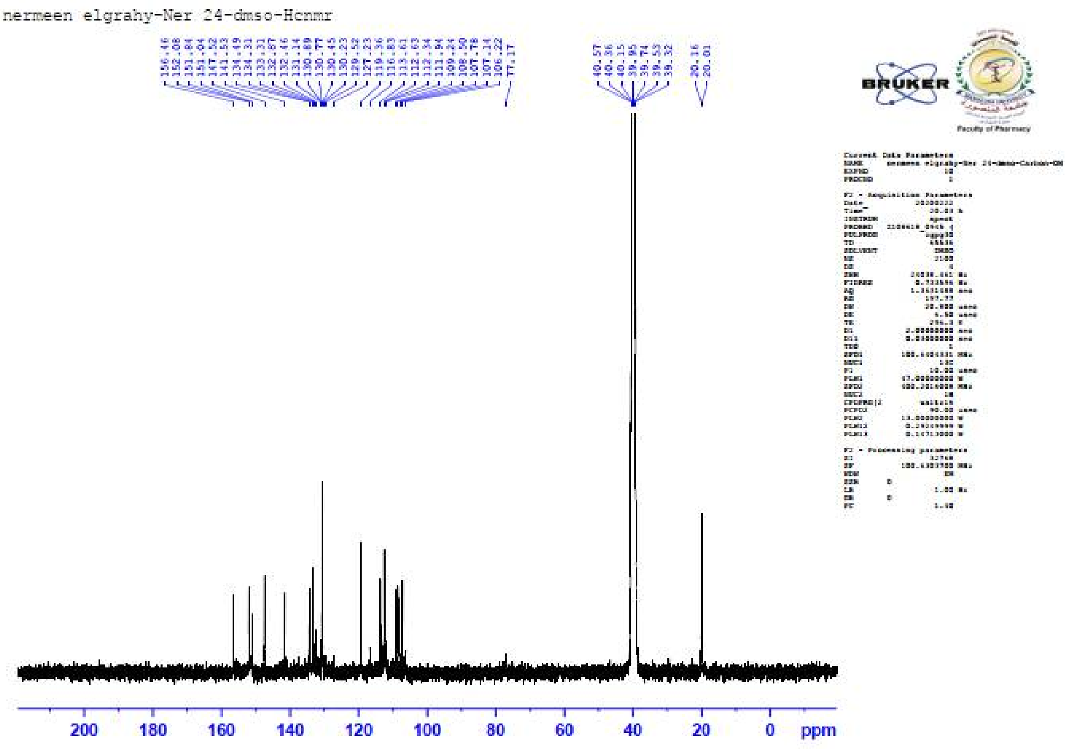

Fig. S9: ^{13}C-NMR of compound **5b**
